# Positive family history of colorectal cancer in a general practice setting [FRIDA.Frankfurt]: study protocol of a of a cross-sectional study

**DOI:** 10.1186/s12885-015-1600-7

**Published:** 2015-08-28

**Authors:** Andrea Siebenhofer, Jasper Plath, Maja Taubenroth, Susanne Singer, Marlene Hechtner, Anne Dahlhaus, Sandra Rauck, Sylvia Schulz-Rothe, Insa Koné, Ferdinand M. Gerlach

**Affiliations:** 1 Institute of General Practice, Goethe-University Frankfurt, Frankfurt am Main, Germany; 2 German Cancer Research Center (DKFZ), Heidelberg, Germany; 3 German Cancer Consortium (DKTK), Heidelberg, Germany; 4 Institute of General Practice and Evidence-based Health Services Research, Medical University of Graz, Graz, Austria; 5 Institute of Medical Biostatistics, Epidemiology and Informatics (IMBEI), Johannes Gutenberg University Mainz, Mainz, Germany

## Abstract

**Background:**

Although the risk of developing colorectal cancer (CRC) is 2-4 times higher in case of a positive family history, risk-adapted screening programs for family members related to CRC- patients do not exist in the German health care system. CRC screening recommendations for persons under 55 years of age that have a family predisposition have been published in several guidelines.

The primary aim of this study is to determine the frequency of positive family history of CRC (1^st^ degree relatives with CRC) among 40–54 year old persons in a general practitioner (GP) setting in Germany. Secondary aims are to detect the frequency of occurrence of colorectal neoplasms (CRC and advanced adenomas) in 1^st^ degree relatives of CRC patients and to identify the variables (*e.g.* demographic, genetic, epigenetic and proteomic characteristics) that are associated with it. This study also explores whether evidence-based information contributes to informed decisions and how screening participation correlates with anxiety and (anticipated) regret.

**Methods/Design:**

Prior to the beginning of the study, the GP team (GP and one health care assistant) in around 50 practices will be trained, and about 8,750 persons that are registered with them will be asked to complete the “Network against colorectal cancer” questionnaire. The 10 % who are expected to have a positive family history will then be invited to give their informed consent to participate in the study. All individuals with positive family history will be provided with evidence-based information and prevention strategies. We plan to examine each participant’s family history of CRC in detail and to collect information on further variables (*e.g.* demographics) associated with increased risk. Additional stool and blood samples will be collected from study-participants who decide to undergo a colonoscopy (*n* ~ 350) and then analyzed at the German Cancer Research Center (DKFZ) Heidelberg to see whether further relevant variables are associated with an increased risk of CRC. One screening list and four questionnaires will be used to collect the data, and a detailed statistical analysis plan will be provided before the database is closed (expected to be June 30, 2015).

**Discussion:**

It is anticipated that when persons with a family history of colorectal cancer have been provided with professional advice by the practice team, there will be an increase in the availability of valid information on the frequency of affected individuals and an increase in the number of persons making informed decisions. We also expect to identify further variables that are associated with colorectal cancer. This study therefore has translational relevance from lab to practice.

**Trial registration:**

German Clinical Trials Register DRKS00006277

**Electronic supplementary material:**

The online version of this article (doi:10.1186/s12885-015-1600-7) contains supplementary material, which is available to authorized users.

## Background

The risk of developing colorectal cancer (CRC) is 2–4 times higher in case of a family predisposition [1, 2]. A repeat occurrence within a family can be noted in around 30 % of all cases of CRC, whereby around 5 % of these are associated with hereditary types of CRC. The family predisposition for the remaining ~25 % of cases has not yet been properly explained [3]. The different constellations of risk are currently not taken into account in the directives of the German Federal Joint Committee on cancer screening [4]. In 2013, the usefulness of colorectal cancer screening for persons under 55 years of age with a family predisposition was declared by the IQWIG to be uncertain, as no high quality studies could be identified in which comprehensive screening strategies in the general population had been analyzed using anamnestic instruments [5]. The “Network against colorectal cancer” questionnaire has shown that information on a positive family history is partly overstated when patients fill in questionnaires themselves, as opposed to when they are personally interviewed, and that when findings are positive, only 40 % of those concerned inform their GP or gastroenterologist [6]. In two current reviews, the role of the GP and other medical personnel is described as being the most important factor influencing the decision to participate in screening examinations [7, 8], and as having a greater influence than written invitations [8]. As around 92 % of the German general population have a GP [9] and around 89 % annually make use of outpatient services [10], screening participation rates are expected to be high. The “European Guideline for Quality Assurance in CRC screening and diagnosis” recommends that patients should be spoken to personally [11]. This is easy for the GP to do on account of the trusting relationship he has with his patients.

In view of the new law on the further development of cancer screening, as well as further clarification demanded by the German Federal Joint Committee, the proposed project could raise the awareness of the importance of family predisposition for future CRC screening programs [12].

## Methods/Design

### Research questions

The following primary research question will be addressed:

- What is the frequency of positive family history of CRC (1^st^ degree relatives with CRC) among 40–54 year old persons in a German GP setting?

Secondary research questions are:

- What is the frequency of colorectal neoplasms (CRC and advanced adenomas) in 1^st^ degree relatives of CRC patients in a German GP setting?
- What variables (e.g., demographic, genetic, epigenetic and proteomic characteristics) are associated with an increased risk of CRC?
- How can evidence-based information contribute to informed decisions with respect to screening?
- How does screening participation correlate with anxiety and regret?

### Study design and setting

FRIDA.Frankfurt is a cross-sectional study in a general practice setting. Prior to the beginning of the study, the practice team (GP and one health care assistant (HCA)) will be briefly trained by study personnel in how to conduct the study in their practices. Before this session, a list of all 40–54 year old patients who have attended the practice within the last twelve months will be compiled by means of the practice software. Afterwards the HCA will contact eligible patients and complete the “Network against colorectal cancer” questionnaire [13] (see screening list, Additional file 1) during a routine practice visit, or per telephone.

Those who have a positive family history will then be invited to participate in the study and to give their informed consent to do so. With the help of study materials that are presented clearly and informatively (both graphic and text based), the GP is expected to be able to provide family members related to CRC patients with adequate evidence-based information and to describe prevention strategies. During this *first practice visit* we plan to examine the participant’s family history of (colorectal-) cancer in further detail, collect information on previous CRC-screening tests, and gather information on other variables (e.g. demographics) associated with an increased risk of CRC (see questionnaire 1, Additional file 2). Study participants with a hereditary risk of CRC (suspected or already known) will be documented and excluded from subsequent study-phases.

In a *second practice visit* within two weeks of the first, details on anxiety, anticipated regret and reasons for or against participation with respect to screening will be collected before the study participants inform their GP about their decision. Furthermore, informed decision will be assessed using the validated instrument from Steckelberg et al. [14]. The selected screening test (colonoscopy, fecal occult blood test) will be documented (see questionnaire 2, Additional file 3).

If study participants are willing to undergo a colonoscopy, the GP will schedule an appointment with the gastroenterologist, and ask the participant for a blood- and stool-sample and to fill in an additional questionnaire (see questionnaire 3, Additional file 4). The results of the colonoscopy will be documented from the gastroenterologist’s report for the GP. We will also collect information on study participants who are not willing to participate in CRC-screening (e.g. colonoscopy before study participation due to a known positive family history of CRC).

**Fig. 1:**
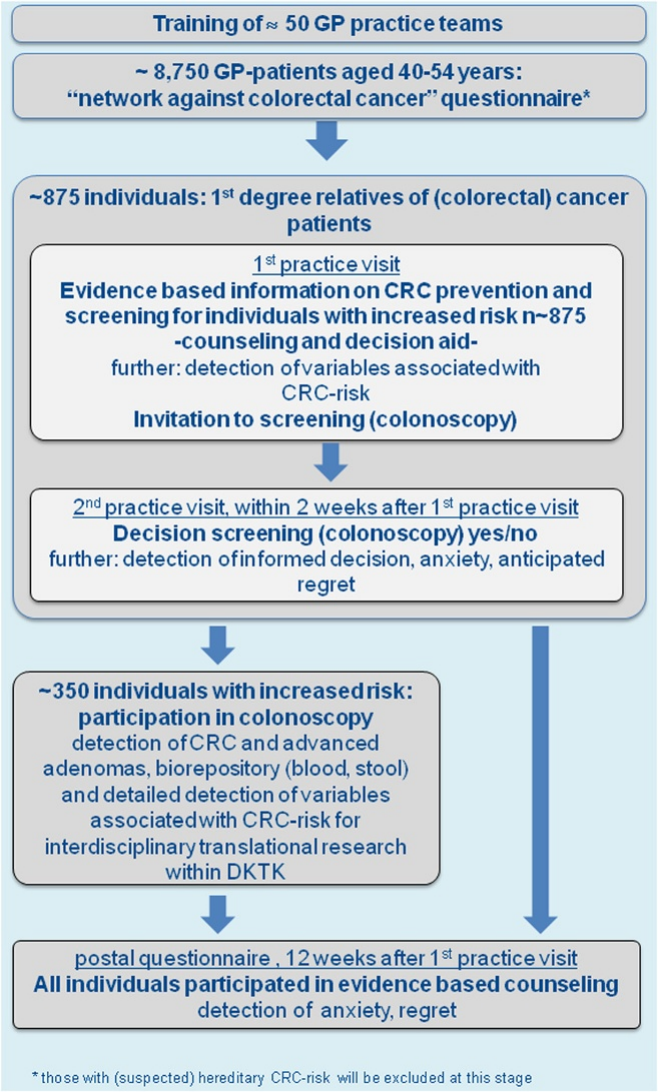
Flow chart of the FRIDA.Frankfurt Study

*Twelve weeks after the first practice visit,* questionnaires asking about anxiety and regret will be sent out by post to all persons who participate in the study (see questionnaire 4, Additional file 5). For further details see flow chart (Fig. 1).

Details on methods and design are laid down in the original study protocol, which can be provided by the corresponding author on demand.

### Main practice and patient in- and exclusion criteria

Doctors at participating trial sites must work as a general practitioner (GP or specialist in internal medicine), provide health services to persons with German statutory health insurance, have software which is capable of detecting potentially eligible patients, and work in a practice located in the German state of Hesse. Participating GPs and Health Care Assistants must also agree to the contractual obligations of the trial.

Patients must be 40–54 years of age, regularly attend the GP’s practice (at least one contact in the last 12 months) and sign an informed consent form. A lack of German language skills and gravidity are exclusion criteria for patients.

### Sample size calculation

As of August 2013, the “Forschungsnetzwerk Allgemeinmedizin Frankfurt” (ForN) [15] database contained approximately 100 general practices. An average-sized practice treats about 1000 patients/quarter of which about 25 % are 40–54 years of age. Over the course of a year, about 250 eligible patients will attend any one practice. It is to be expected that at least 50 % of practices (50) will participate in the study and at least 70 % of patients will complete the questionnaire (175/practice). The expected sample size is at least 8,750 of which about 875 (10 %) are expected to have a positive family history of colorectal cancer [16]. Of these, we expect around 350 (40 %) persons to follow the invitation to have a colonoscopy.

### Recruitment, study timeline and reimbursement

**Fig. 2:**
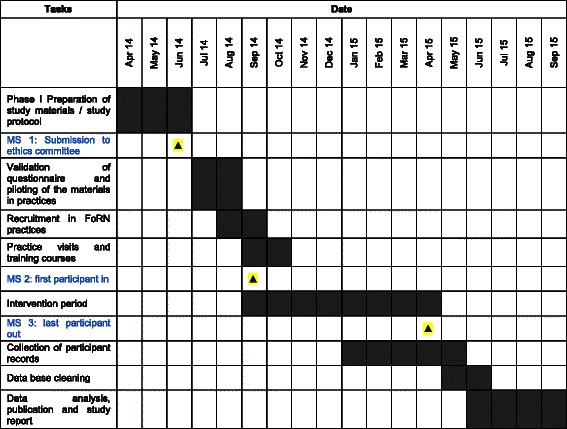
Timeline of the of the FRIDA.Frankfurt Study

The trial will be primarily conducted in general practices in the state of Hesse. Eligible practices will be recruited mainly through the “Forschungsnetzwerk Allgemeinmedizin Frankfurt” (ForN) which is a network of general practices that have successfully conducted research projects with our Institute [15]. During an approximately eight-month period beginning in September, 2014, about 8,750 persons attending the general practices will be asked about their family history (see Fig. 2). As compensation, recruitment practices will receive €2 per individual that is interviewed by the HCA on the basis of the “network against colorectal cancer questionnaire” / screening list, and €10 per individual that is counselled by a GP, provided with the evidence-based decision aid, and completed all questionnaires on risk-factors, anxiety, (anticipated) regret and informed decision.

### Data collection and quality assurance/avoidance of biases and monitoring

At the practices, the HCA will contact eligible patients and complete the “Network against colorectal cancer” questionnaire in a screening list with them (see screening list, Additional file 1). Every document includes information on how to fill in the form. The informed consent forms are sent to the Institute of General Practice (IGP) via fax on the day of the participant’s visit to the practice, and the questionnaires will later be collected from the practice by a member of the study team. Questionnaire 4 will be send to the study-participants by post. If necessary, the IGP will send out a reminder to participants after 2 weeks. All questionnaires are shown in Additional files 2, 3, 4 and 5.

The IGP study team will ensure that all processes in the trial comply with Good Clinical Practice (GCP) guidelines, the legal requirements and the standard operating procedures (SOPs) of the IGP. The patient questionnaires, patient information brochure and informed consent form will be piloted to check their comprehensibility and applicability. During the first visit, participating GPs and the HCA will be thoroughly trained and provided with the information required to conduct all steps in the study. Amongst other things, this will include the identification of suitable patients, help for patients in filling out the questionnaire on socio-demographic data, and the collection of data on those that are not willing to participate.

All information from forms (e.g. CRFs) will be transferred to the clinical study database (IBM SPSS Statistics). A data check of this database will take place according to pre-defined trial rules (range-, validity-, and consistency- checks according to defined SOPs developed during the course of the trial and documented in the trial master file). Follow-up enquiries resulting from the data plausibility check will be resolved with the help of the GP or HCA concerned.

The collection and processing of patient data will always be conducted using the patient identification number (Pat.-ID) pseudonym from the GP’s practice software. To ensure high-quality data, all pseudonymization will be conducted by the trained HCA in the GP’s practice.

For the future investigation of genetic, epigenetic and proteomic biomarkers associated with CRC-risk, a blood and stool sample will be taken from study-participants that have decided to undergo a colonoscopy. The blood and stool samples and data from questionnaires about other risk-factors will be transferred to the German Cancer Research Center (DKFZ) in pseudonymized form and stored there. In addition, a qualitative and a quantitative fecal occult blood test will be performed at the DKFZ. All blood and stool samples, as well as further data, will be anonymized at the DKFZ and stored in the central biorepository.

Study members of the IGP will perform on-site monitoring visits as frequently as necessary and record the dates of their visits in a database (Access®). At the visits, a study member will check the completeness and accuracy of the data, and, if necessary, compare the data entered into the CRFs with clinical records (source documents). Direct access to source documents must be permitted in order to verify that the data recorded in the CRF and other forms are consistent with the original source data. Findings from this review will be discussed with the GP and HCA. The study members will stay in regular contact with the GP and HCA and provide feedback on the course of the study.

Data collection is scheduled to be completed by June 30, 2015.

### Analysis

The data collected in this study will be described in terms of mean, standard deviation, median, minimum, maximum and quartiles for continuous variables, while absolute and relative frequencies will be computed for categorical variables. Scores on the applied standardized questionnaires will be calculated according to the user manuals. Frequencies of positive family histories will be reported in combination with confidence intervals.

Exploration of associated variables and group comparisons, e.g. in patients that have undergone or not undergone a colonoscopy, will be performed using logistic or linear regression models as appropriate. All models will be adjusted for relevant confounders. Mixed linear models will be applied in case of paired data or repeated measures.

A detailed description of the statistical methods used in this study will be provided in a Statistical Analysis Plan (SAP) which will be finished before database closure.

### Ethical approval and study registration

Ethical approval for the study was obtained from the leading Ethics Committee at the Frankfurt University Hospital on July 8, 2014. Once the ethical approval has been given by one ethical council in Germany, it applies to all participating sites in the same federal state. The study has been registered in the German Clinical Trials Register; DRKS00006277 [17].

## Discussion

In this cross-sectional study we primarily want to investigate the frequency of positive family history of CRC (1^st^ degree relatives with CRC) among 40–54 year old persons in a German GP setting. Further outcomes are to detect the frequency of colorectal neoplasms (CRC and advanced adenomas) in 1^st^ degree relatives of CRC patients and the kinds of variables that are associated with an increased risk of CRC. In addition, one major objective will be to measure how evidence-based information contributes to making informed decisions with respect to screening and how participation in screening correlates with anxiety and (anticipated) regret.

The role of the GP is an important factor towards screening examinations [7, 8]. It would therefore appear reasonable to expect data that is collected by a specially trained and therefore highly motivated general practice team to be of significantly higher quality. It can further be expected that when professional advice is provided by such a general practice team, colonoscopy participation rates will be higher than otherwise [18], and that this, in turn, will result in an increase in the size of the cohort available to collect information on variables (both demographic variables and biomarkers) for further identification of persons with a positive medical family history. The absence of standardized colonoscopy reports from different gastroenterologists is a potential limitation of this study. Further, variables associated with an increased risk of CRC will be analyzed only among 1^st^ degree relatives of CRC patients. However, this study provides a strong translational context as it involves the creation of new collaborations and synergies involving GPs, clinical epidemiologists and gastroenterologists.

## Additional files

Additional file 1: **Screening list.** (PDF 85 kb)
Additional file 2: **Questionnaire 1.** (PDF 286 kb)
Additional file 3: **Questionnaire 2.** (PDF 256 kb)
Additional file 4: **Questionnaire 3.** (PDF 244 kb)
Additional file 5: **Questionnaire 4.** (PDF 225 kb)

## Abbreviations

CRC: Colorectal cancer
CRF: Case Report Form
DKFZ: German Cancer Research Center
GP: General practitioner
HCA: Health care assistant
IGP: Institute of General Practice
IQWiG: Institute for Quality and Efficiency in Health Care
SOP: Standard operating procedure
