# Supplementary material for: Positive family history of colorectal cancer in a general practice setting [FRIDA.Frankfurt]: study protocol of a of a cross-sectional study
Source: BMC Cancer. 2015 Aug 28;15:605. doi: 10.1186/s12885-015-1600-7 (PMC4552264; doi:10.1186/s12885-015-1600-7)
Supplement: Additional file 1: — Screening list. (PDF 85 kb) [file 12885_2015_1600_MOESM1_ESM.pdf]

Pleae ask every patient on the list the following four questions:

Question 1: A **first-degree relative** of mine (parents, siblings, children) developed bowel cancer.

Question 2: A **first-degree relative** of mine (parents, siblings, children) who developed bowel cancer was **under 50 years of age**.

Question 3 A **bowel polyp** (adenoma) was discovered in a **first-degree relative** of mine (parents, siblings, children) before his or her 50th birthday.

Question 4 **Three or more relatives** of mine developed bowel cancer, stomach cancer, uterine cancer, ovarian cancer, ureteral cancer or cancer of the renal pelvis.

....

Patient-ID<br><br>Patient's date of birth:<br>______.______.__________		➡	**Were you able to ask the patient the four questions about hereditary risk?**<br>☐ Yes, in the practice<br>☐ Yes, on the phone<br>☐ No, no interest<br>☐ Knows about hereditary risk (please ask questions and invite patient anyway)<br>☐ No, couldn't reach patient after 3 attempts<br>☐ No, because____________________	➡	**The answers to the 4 questions:**<br>Question 1: ☐ No ☐ Yes→→→<br>Question 2: ☐ No ☐ Yes→→→<br>Question 3: ☐ No ☐ Yes→→→<br>Question 4: ☐ No ☐ Yes→→→	➡	**Arranged consultation?**<br>☐ Yes, forwarded patient directly to doctor<br>☐ Yes, arranged consultation<br>☐ Patient desires further contact<br>☐ No, because:________________
Gender:<br>☐ female<br>☐ male	Date:<br>______.______.__________						**Did the consultation with the doctor take place?**<br>☐ Yes<br>☐ No, because________________

Patient-ID<br><br>Patient's date of birth:<br>______.______.__________		➡	**Were you able to ask the patient the four questions about hereditary risk?**<br>☐ Yes, in the practice<br>☐ Yes, on the phone<br>☐ No, no interest<br>☐ Knows about hereditary risk (please ask questions and invite patient anyway)<br>☐ No, couldn't reach patient after 3 attempts<br>☐ No, because____________________	➡	**The answers to the 4 questions:**<br>Question 1: ☐ No ☐ Yes→→→<br>Question 2: ☐ No ☐ Yes→→→<br>Question 3: ☐ No ☐ Yes→→→<br>Question 4: ☐ No ☐ Yes→→→	➡	**Arranged consultation?**<br>☐ Yes, forwarded patient directly to doctor<br>☐ Yes, arranged consultation<br>☐ Patient desires further contact<br>☐ No, because:________________
Gender:<br>☐ female<br>☐ male	Date:<br>______.______.__________						**Did the consultation with the doctor take place?**<br>☐ Yes<br>☐ No, because________________

....

Design: Spiral binding,

Size: Four entries (Participants) per site, overall 250 entries, around 70 pages

Institut für Allgemeinmedizin, Goethe-Universität Frankfurt am Main;
Studiensekretariat: ☎ 069-6301-7152 🖷 069-6301-6428
💻 frida-studie@allgemeinmedizin.uni-frankfurt.de
