# Supplementary material for: Positive family history of colorectal cancer in a general practice setting [FRIDA.Frankfurt]: study protocol of a of a cross-sectional study
Source: BMC Cancer. 2015 Aug 28;15:605. doi: 10.1186/s12885-015-1600-7 (PMC4552264; doi:10.1186/s12885-015-1600-7)
Supplement: Additional file 2: — Questionnaire 1. (PDF 286 kb) [file 12885_2015_1600_MOESM2_ESM.pdf]

FRIDA.Frankfurt
Familiäres Risiko für Darmkrebs

Questionnaire 1

(To be filled out by the family doctor, or HCA and study participant together)

**Date**	
**Patient-ID**	

Dear Colleagues, dear Practice Team,

The aim of this questionnaire, which will take 10-15 minutes to fill out, is to gather information on trial participants. **You and the study participant* should fill it out together.** Please pay attention to the following instructions:

- The questions in this questionnaire are directed towards the trial participants.
- Please clearly tick the appropriate box. If you make a mistake, cross out the corresponding box and tick the correct one. **Any changes should be dated and signed or initialed**.
- **Please answer every question.** If you are in any doubt, you should tick the box which is most appropriate.
- Your answers will of course be treated in **strict confidence**. Only pseudonymized data will be used in the analysis.

If you have any questions, please do not hesitate to contact us. You will find contact details below. **Thank-you!**

*The masculine form is used for both genders to facilitate readability.

This page is to be filled out by the family doctor/HCA:

**1.**	**Please enter gender of trial participant.**<br><br>☐ female<br>☐ male
**2.**	**Please enter date of birth of trial participant.**<br><br>|_|_|. |_|_|.|_|_|_|_|

**3.**	**Please enter the body height of the trial participant in cm.**<br><br>|_|_|_| **cm**
**4.**	**Please enter the body weight of the trial participant in kg.**<br><br>|_|_|_| **kg**

The following questions should be answered by the trial participants:

Questions on health

5. How often do you visit your family doctor annually?

On average around |_|_| times a year

6. Did you gather information on the existence of cancer in your family before answering this questionnaire? *(e.g. by asking relatives)*

☐ No

☐ Yes

7. Before this consultation, did you know that you have an increased risk of developing bowel cancer?

☐ No

☐ Yes

8. Have you ever gathered information on bowel cancer screening?

☐ No

☐ Yes, from ______________________________

9. Please evaluate the following statements?
(Please cross one box for each statement)

**I would like to receive information on bowel cancer screening from …**	Strongly agree	Somewhat agree	Somewhat disagree	Strongly disagree
…my **family doctor**	☐	☐	☐	☐
…my **health insurance**	☐	☐	☐	☐
…a **public institution** (e.g. the Office of Public Health)	☐	☐	☐	☐
…a **private organization** (e.g. self-help groups, associations)	☐	☐	☐	☐
…the **internet**	☐	☐	☐	☐
… **newspapers, magazines**	☐	☐	☐	☐
…the **television**	☐	☐	☐	☐

10. How would you like to see the bowel cancer screening procedure?
(Please cross one box for each statement)

	Strongly agree	Somewhat agree	Somewhat disagree	Strongly disagree
**My family doctor should decide** whether it is best that I undergo bowel cancer screening.	☐	☐	☐	☐
My family doctor should provide me with information on the risks and benefits of bowel cancer screening, so that **I can decide what is best for me**.	☐	☐	☐	☐

11. Have you had a colonoscopy?

☐ No

☐ Yes

<u>If yes:</u>

When was the last one? |_|_|_|_| *(year)*

How often? |_|_| times

Were bowel polyps found?

☐ No ☐ Don't know ☐ Yes

Why was the last colonoscopy carried out?
(Please tick the reasons.)

☐ fecal occult blood test was positive
☐ blood in the stool was visible
☐ changes in bowel habits (e.g. diarrhea, constipation, consistency)
☐ persistent stomach pain/cramps
☐ other complaints
☐ known family history of bowel cancer

12. **Have you ever been diagnosed with cancer?**

☐ No

☐ Yes

If so, what type of cancer?

1. ______________________________ for the first time aged |_|_| years

2. ______________________________ for the first time aged |_|_| years

3. ______________________________ for the first time aged |_|_| years

Questions about your parents:

13. **When was your mother / father born?**
(only "biological parents")

Mother: |_|_|_|_| *year of birth* ☐ Don't know

Father: |_|_|_|_| *year of birth* ☐ Don't know

14. **Is your mother / father still alive?**
(only "biological parents")

Mother: ☐ No ☐ Yes ☐ Don't know

Father: ☐ No ☐ Yes ☐ Don't know

If not, when did she/he die (year)?

Mother: |_|_|_|_|

Father: |_|_|_|_|

15. Did your parents have bowel cancer?
(only "biological parents")

Mother: ☐ No ☐ Yes, aged |_|_| years ☐ Don't know

Father: ☐ No ☐ Yes, aged |_|_| years ☐ Don't know

16. Did you mother have <u>another type of cancer</u>?
(only "biological mother")

☐ No ☐ Yes ☐ Don't know

<u>If so, what type(s) of cancer were they?</u>

1.______________________________ for the first time aged |_|_| years

2.______________________________ for the first time aged |_|_| years

3.______________________________ for the first time aged |_|_| years

17. Did you father have <u>another type of cancer</u>?
(only "biological father")

☐ No ☐ Yes ☐ Don't know

<u>If so, what type(s) of cancer were they?</u>

1.______________________________ for the first time aged |_|_| years

2.______________________________ for the first time aged |_|_| years

3.______________________________ for the first time aged |_|_| years

Questions about siblings

18. Do you or did you have siblings?
(only "biological siblings" incl. half brothers and sisters)

☐ No → if not, please go to question 22

☐ Yes

19. How many sisters / brothers do you or did you have?
(only "biological siblings" incl. half brothers and sisters)

Sisters: |_|_| Brothers: |_|_|

20. Have any of your brothers /sisters had bowel cancer?
(only "biological siblings" incl. half brothers and sisters)

☐ No

☐ Don't know

☐ Yes

If so:

How many of your sisters/brothers? |_|

Please fill out one field for every brother or sister that has had bowel cancer.

One sister/brother
She/he was |_|_| years old when diagnosed with bowel cancer

Another sister/brother
She/he was |_|_| years old when diagnosed with bowel cancer

Another sister/brother
She/he was |_|_| years old when diagnosed with bowel cancer

Another sister/brother
She/he was |_|_| years old when diagnosed with bowel cancer

21. Has one or your sisters / one of your brothers ever had <u>another type of cancer</u>?
(only "biological siblings" incl. half brothers and sisters)

☐ No
☐ Don't know
☐ Yes

<u>If so:</u>

How many of your sisters/brothers? |_|

<u>What type(s) of cancer were they?</u>

One sister/one brother had: *(what type of cancer?)*

______________________________ for the first time aged |_|_| years

Another sister/another brother had: *(what type of cancer?)*

______________________________ for the first time aged |_|_| years

Another sister/another brother had: *(what type of cancer?)*

______________________________ for the first time aged |_|_| years

Another sister/another brother had: *(what type of cancer?)*

______________________________ for the first time aged |_|_| years

Another sister/another brother had: *(what type of cancer?)*

______________________________ for the first time aged |_|_| years

22. Have any of your <u>second-degree</u> relatives ever had bowel cancer?
(only "biological relatives")
Grandmother, Grandfather, Uncle, Aunt, Cousin:

☐ No
☐ Don't know
☐ Yes

<u>If so:</u>

How many of your second-degree relatives? |_|_|
(Grandmother, Grandfather, Uncle, Aunt, Cousin)

How old was your relative when diagnosed with bowel cancer. If several relatives had bowel cancer, please enter the age of the relative that was youngest at the time of the diagnosis.

|_|_| years

For example: Your uncle fell ill when he was 60 and your grandmother when she was 48. In this case, please enter the age of your grandmother i.e. 48 years.

23. Have any of your <u>second-degree</u> relatives had <u>another type of cancer</u>?
(only "biological relatives")
Grandmother, Grandfather, Uncle, Aunt, Cousin

☐ No
☐ Don't know
☐ Yes

<u>If so:</u>

How many of your second-degree relatives? |_|_|
(Grandmother, Grandfather, Uncle, Aunt, Cousin)

<u>What type(s) of cancer were they?</u>

One relative had: *(What type of cancer?)*

______________________________ for the first time aged |_|_| years

Another relative had: *(What type of cancer?)*

______________________________ for the first time aged |_|_| years

Another relative had: *(What type of cancer?)*

______________________________ for the first time aged |_|_| years

Questions about the person and living situation

24. Your nationality?

☐ German

☐ another ______________________________

25. What country were you born in?

____

26. Since when have you lived in Germany? (including the former GDR)

☐ since birth

☐ since |_|_|_|_| (year)

27. What country were your parents born in?

Mother: ______________________________

Father: ______________________________

28. How many people live in your household?

(Please count yourself and children too)

A total of |_|_| persons

→How many of those persons are under 15? |_|_|

29. What school leaving qualification do you have?

(Please tick one box)

☐ Finished school without a qualification

☐ Graduated from ninth grade in secondary school

☐ Graduated from tenth grade in secondary school

☐ Entrance qualification for University of Applied Science

☐ Higher education entrance qualification / baccalaureate

☐ Other school graduation certificate

30. What is your highest vocational training or university qualification?

(Please tick one box only)

☐ No professional qualification

☐ Apprenticeship (vocational/operational training)

☐ Vocational / commercial school

☐ Professional school (e.g. technician)

☐ University of Applied Science

☐ University

☐ Another qualification

31. Which of the following is true of your employment situation?

(Please tick one box only)

Currently I am…

☐ …in full-time employment

☐ …in part-time employment

☐ …employed occasionally or irregularly

☐ …not employed (pensioner / housewife etc.)

☐ …seeking a job

32. Are you the main earner in your household?

☐ No

☐ Yes

33. What is your current employment situation, or where were you most recently employed?
(Please tick the most appropriate box in the category which best describes your situation.)

Blue collar employee
- ☐ unskilled worker
- ☐ skilled worker
- ☐ craftsman
- ☐ foreman
- ☐ master craftsman
- ☐ unspecified

White-collar employee
- ☐ job involves following general instructions *(e.g. cashier, receptionist etc.)*
- ☐ skilled job that involves following instructions *(e.g. clerk, salesperson, technical draftsman)*
- ☐ independent position or position with management responsibility for personnel *(e.g. junior researcher, authorized signatory, department head)*
- ☐ position with substantial management and decision-making responsibilities *(e.g. director, executive, member of board of management)*
- ☐ unspecified

Civil servant (also professional soldier / judicial officer)
- ☐ low level *(up to level of service manager)*
- ☐ medium level *(chief secretary, judicial officer)*
- ☐ higher level *(from inspector to second secretary)*
- ☐ high level, judge, professor etc. *(from councilor upwards)*
- ☐ unspecified

Agriculture
- ☐ utilized agricultural land below 10 hectares
- ☐ utilized agricultural land 10 hectares and more
- ☐ member of agricultural cooperative
- ☐ unspecified

Member of professional classes (e.g. doctor, lawyer, tax advisor etc.)
- ☐ self-employed
- ☐ 1-4 employees
- ☐ 5 or more employees
- ☐ unspecified

Self employed in trade, commerce and commercial services
- ☐ self-employed
- ☐ 1-4 employees
- ☐ 5 or more employees
- ☐ Member of manufacturing cooperative
- ☐ unspecified

Family business
- ☐ assisting family members

Other (please specify)

____

34. How high is your household's total monthly net income?

Total monthly net income is understood to mean wages and salaries, income from self employment, pensions and annuities, public sector support, income from renting/leasing, housing allowance and other income. Taxes and social insurance contributions should be deducted from the total.

You can rest assured that it will not be possible to associate names with answers when the data is evaluated!

(Please tick only one box.)

☐ below € 1,250

☐ € 1,250 to below € 1,750

☐ € 1,750 to below € 2,250

☐ € 2,250 to below € 3,000

☐ € 3,000 to below € 4,000

☐ € 4,000 to below € 5,000

☐ more than € 5,000

Thank-you for participating!

Sources:

Questions 28,29,30,32,33,34: Based on Lampert T, Kroll L. Messung des sozioökonomischen Status in sozialepidemiologischen Studien. In: Richter M, Hurrelmann K, editors. Gesundheitliche Ungleichheit: VS Verlag für Sozialwissenschaften; 2006. p. 297-319.
