# Supplementary material for: Positive family history of colorectal cancer in a general practice setting [FRIDA.Frankfurt]: study protocol of a of a cross-sectional study
Source: BMC Cancer. 2015 Aug 28;15:605. doi: 10.1186/s12885-015-1600-7 (PMC4552264; doi:10.1186/s12885-015-1600-7)
Supplement: Additional file 3: — Questionnaire 2. (PDF 256 kb) [file 12885_2015_1600_MOESM3_ESM.pdf]

FRIDA.Frankfurt

Familiäres Risiko für Darmkrebs

Questionnaire 2

(The following is to be completed by the family practice)

**Date**	
**Patient-ID**	

Dear participant,

Thank-you very much for taking the time to answer the following questions. The questionnaire will only take about ten minutes to fill in. Please observe the following instructions:

- Please clearly tick the appropriate box. If you make a mistake, cross out the corresponding box and tick the correct one.
- **Please answer every question.** If you are in any doubt, you should tick the box which is most appropriate.
- Your answers will of course be treated in **strict confidence**. Only pseudonymized data will be used in the analysis.

If you have any questions, please do not hesitate to contact us. You will find contact details below. **Thank-you!**

Institut für Allgemeinmedizin, Goethe-Universität Frankfurt am Main
Studiensekretariat: ☎ 069-6301-7152 🖷 069-6301-6014
💻 frida-studie@allgemeinmedizin.uni-frankfurt.de

Questions about bowel cancer screening

1. If I don't undergo bowel cancer screening I will regret it at a later date.

☐ Yes
☐ No

2. I will participate in bowel cancer screening by having an occult blood test.

☐ Yes
☐ No

3. I will participate in bowel cancer screening by having a colonoscopy.

☐ Yes
☐ No

Questions about feelings and emotions

4. Over the last two weeks, how often have you been bothered by any of the following problems?

Please tick the appropriate answer with „✓".

Over the last two weeks	Not at all	Several days	More than half the days	Nearly every day
1. Feeling nervous, anxious or on edge	0	1	2	3
2. Not being able to stop or control worrying	0	1	2	3

5. Over the last two weeks, how often have you been bothered by any of the following problems?

Please tick the appropriate answer with **„✓“.**

**Over the last two weeks**	**Not at all**	**Several days**	**More than half the days**	**Nearly every day**
1. Little interest or pleasure in doing things	0	1	2	3
2. Feeling down, depressed, or hopeless	0	1	2	3
3. Trouble falling or staying asleep, or sleeping too much	0	1	2	3
4. Feeling tired or having little energy	0	1	2	3
5. Poor appetite or overeating	0	1	2	3
6. Feeling bad about yourself – or that you are a failure or have let yourself or your family down	0	1	2	3
7. Trouble concentrating on things, such as reading the newspaper or watching television	0	1	2	3
8. Moving or speaking so slowly that other people could have noticed? Or the opposite – being so fidgety or restless that you have been moving around a lot more than usual?	0	1	2	3
9. Thoughts that you would be better off dead, or of hurting yourself in some way	0	1	2	3

Please read every statement carefully and tick the box which most accurately reflects **your attitude**.

6. I regard participation in bowel cancer screening examinations as:

	1	2	3	4	
reassuring	☐	☐	☐	☐	alarming
important	☐	☐	☐	☐	unimportant
a good thing	☐	☐	☐	☐	a bad thing
an advantage	☐	☐	☐	☐	a disadvantage

Questions about consultations on the familial risk of bowel cancer

In order to answer the questions correctly, please give only one answer. If you are uncertain, please tick the box representing the answer that is most probably correct.

7. The occult blood test is used in screening for which disease (e.g. hemoccult test)?

☐ irritable bowel syndrome
☐ inflammatory bowel disease
☐ bowel cancer
☐ hemorrhoids

8. **What could explain why the result of an occult blood test is false positive?**

When on the day before the test…

- ☐ …the test person ate raw meat
- ☐ …the test person ate fish
- ☐ …the test person drank alcohol
- ☐ …the test person smoked cigarettes

Imagine 1000 people aged 50 years whose occult blood test result turned out to be positive.

9. **How many of the thousand people would you expect to actually have bowel cancer?**

- ☐ 1000 persons
- ☐ 980 persons
- ☐ 700 persons
- ☐ 400 persons
- ☐ 100 persons
- ☐ 10 persons

Please anwer the following question according to your gender. Women only the left question and men only the right question.

Question for women

10a **How many women aged 50 years with familial risk will develop bowel cancer within the next 10 years.**

- ☐ **5** in 1.000 women
- ☐ **12** in 1.000 women
- ☐ **30** in 1.000 women
- ☐ **63** in 1.000 women
- ☐ **122** in 1.000 women

Question for men

10b **How many men aged 50 years with familial risk will develop bowel cancer within the next 10 years.**

- ☐ **8** in 1.000 men
- ☐ **12** in 1.000 men
- ☐ **20** in 1.000 men
- ☐ **63** in 1.000 men
- ☐ **122** in1.000 men

Bowel cancer screening by means of the occult blood test reduces the risk of dying of bowel cancer.
Assuming 1000 people annualy undergo bowel cancer screening.

11. How many fewer people will die of bowel cancer?

- ☐ 2-3 persons
- ☐ 8-10 persons
- ☐ 50-100 persons
- ☐ 200-400 persons
- ☐ 800-990 persons

An occult blood test or a colonoscopy can be carried out in order to screen for bowel cancer. It is important that studies are undertaken to ascertain the risks and benefits of such medical procedures.

12. Do you think such studies exist for people <u>without</u> positive family history of bowel cancer?

Colonoscopy	☐ Yes	☐ No
Occult blood test	☐ Yes	☐ No

13. Do you think such studies exist for people <u>with</u> positive family history of bowel cancer?

Colonoscopy	☐ Yes	☐ No
Occult blood test	☐ Yes	☐ No

	Imagine 1000 people aged 50 years whose occult blood test result turned out to be negative.
**14.**	**In your opinion, how many of the thousand people do not have bowel cancer?**
	☐ 1000 persons<br>☐ 999 persons<br>☐ 990 persons<br>☐ 850 persons<br>☐ 400 persons<br>☐ 100 persons<br>☐ 10 persons
	A colonoscopy can have severe side effects.
**15.**	**Which of the following side effects can occur as the result of a colonoscopy?**<br>**There are several correct answers to this question. Please tick all the correct answers.**
	☐ fecal incontinence<br>☐ internal bleeding<br>☐ bowel obstruction<br>☐ bowel rupture<br>☐ death

Questions about your reasons for undergoing or not undergoing bowel cancer screening

**16.**	**What are the arguments in favor of undertaking bowel cancer screening in your case?**
	(Please tick every correct answer, regardless of how many there are)
	☐ to know for certain whether I have bowel cancer or not<br>☐ the possibility to detect bowel cancer at an early stage<br>☐ to watch over my health<br>☐ the fear of having bowel cancer<br>☐ as a result of complaints<br>☐ because the risk of my dying of bowel cancer will be reduced<br>☐ other reasons

17. What are the arguments against undertaking bowel cancer screening in your case?

(Please tick every correct answer, regardless of how many there are)

- ☐ no complaints
- ☐ I am afraid bowel cancer may be detected
- ☐ I don’t want to know whether I have bowel cancer
- ☐ I have no time for it
- ☐ I have more serious and important health problems
- ☐ I am afraid of the risks of the screening examination
- ☐ other reasons

18. Did you answer this questionnaire with the help of the information leaflet on the familial risk of developing bowel cancer that your family doctor gave you during your consultation?

- ☐ Yes
- ☐ No

Thank-you for your participation

Sources:

Questions 6-15:: Mit freundlicher Genehmigung von Anke Steckelberg, MIN Fakultät, Universität Hamburg

Question 4: GAD-2: Kroenke K, Spitzer RL, Williams JBW. The PHQ-9. Validity of a brief depression severity measure. J Gen Intern Med 2001; 16: 606–613

Question 5: PHQ-9: Kroenke K, Spitzer RL, Williams JBW, Monahan PO, Löwe B. Anxiety disorders in primary care: prevalence, impairment, comorbidity, and detection. Ann Intern Med 2007; 146: 317-325
