# Supplementary material for: Positive family history of colorectal cancer in a general practice setting [FRIDA.Frankfurt]: study protocol of a of a cross-sectional study
Source: BMC Cancer. 2015 Aug 28;15:605. doi: 10.1186/s12885-015-1600-7 (PMC4552264; doi:10.1186/s12885-015-1600-7)
Supplement: Additional file 4: — Questionnaire 3. (PDF 244 kb) [file 12885_2015_1600_MOESM4_ESM.pdf]

FRIDA.Frankfurt
Familiäres Risiko für Darmkrebs

Questionnaire 3

(only to be filled out when submitting blood and stool sample)

This box will be filled out by the practice team	
**Date**	
**Patient-ID**	
**Barcode on labels**	

Dear participant,

We are pleased that you have decided to submit a blood and stool sample. The aim of the following questionnaire is to gain further information on the risks of developing bowel cancer. It will take 10-15 minutes to fill out. Please pay attention to the following instructions:

- Please clearly tick the appropriate box. If you make a mistake, cross out the corresponding box and tick the correct one.
- **Please answer every question.** If you are in any doubt, you should tick the box which is most appropriate.
- Your answers will of course be treated in **strict confidence**. Only pseudonymized data will be used in the analysis.

If you have any questions, please do not hesitate to contact us. You will find contact details below. **Thank-you!**

1. Has a doctor every diagnosed that you have any of the following diseases?
(Please tick yes or no for every disease. If the answer is yes, how old were you when the illness was diagnosed for the first time?)

Heart attack	☐ No	☐ Yes, aged |_|_|
Decompensated heart failure, angina pectoris	☐ No	☐ Yes, aged |_|_|
Cardiac insufficiency, heart failure	☐ No	☐ Yes, aged |_|_|
Stroke	☐ No	☐ Yes, aged |_|_|
Impaired cerebral blood flow	☐ No	☐ Yes, aged |_|_|
Impaired blood circulation in the legs	☐ No	☐ Yes, aged |_|_|
High blood pressure (hypertension)	☐ No	☐ Yes, aged |_|_|
Elevated blood lipids (e.g. cholesterol)	☐ No	☐ Yes, aged |_|_|
Diabetes (Diabetes mellitus)	☐ No	☐ Yes, aged |_|_|
Gout	☐ No	☐ Yes, aged |_|_|
Gallstones	☐ No	☐ Yes, aged |_|_|
Kidney stones	☐ No	☐ Yes, aged |_|_|
Stomach ulcer/ Duodenal ulcer (Ulcus)	☐ No	☐ Yes, aged |_|_|
Infection with the stomach bacterium *helicobacter pylori*	☐ No	☐ Yes, aged |_|_|
Cataracts	☐ No	☐ Yes, aged |_|_|
Green star (glaucoma)	☐ No	☐ Yes, aged |_|_|
Asthma	☐ No	☐ Yes, aged |_|_|
Hay fever	☐ No	☐ Yes, aged |_|_|
Neurodermitis (atopic eczema, endogenous eczema)	☐ No	☐ Yes, aged |_|_|
Femoral neck fracture	☐ No	☐ Yes, aged |_|_|
Depression	☐ No	☐ Yes, aged |_|_|

2. What medicines do you take on a regular basis?
(Long-term medication)

e.g. Metoprolol-Ratiopharm

The following question is only applicable to women (Men should jump to the next question):
Hormone replacement therapies (e.g. estrogens) can be used to treat menopausal complaints and to prevent certain diseases (e.g. osteoporosis). Some women receive these preparations after their ovaries have been removed.

3. Have you ever had hormone replacement therapy, as prescribed to you by a doctor? (tablets, plasters, gel, injections, cream, drops, spray etc.)

This question does not concern herbal supplements or homeopathic medicines

☐ No

☐ Yes

If so:

For how long in all? |_|_| years

Are you taking such a product at the moment?

☐ Yes What product?______________________________

☐ No When did you last take one? |_|_|_|_| *(year)*

4. Have you ever smoked regularly?

(daily for more than 1 year)

☐ No → If not, please go to question 7

☐ Yes

<u>If so:</u>

Do you still smoke?

☐ No

☐ Yes, on average I smoke on a daily basis

|_|_| cigarettes |_|_| cigars |_|_| pipes

(Please enter zero for the tobacco products that you do not smoke)

5. How old were you when you started to smoke regularly?

|_|_| years of age

If you do not smoke any more:

How old were you when you stopped? |_|_| years of age

6. For how many years did you smoke regularly?

In all around |_|_| years

How much did you smoke on average during this period?

|_|_| cigarettes |_|_| cigars |_|_| pipes

(Please enter zero for the tobacco products that you do not smoke)

7. On how many days a week have you generally drunk alcohol over the last 12 months?

|_| days a week

8. On average, how many alcoholic drinks have you drunk per week over the last 12 months?

(Please enter a zero for those beverages that you did not drink)

**Beer**	**Wine / Sparkling wine**	**Spirits**
(500 ml bottlesl)	*(250 ml glasses)*	*(20 ml glasses)*
|_|_|	|_|_|	|_|_|
bottles/week	*glasses/week*	*glasses/week*

On average per week over the last 12 months

9. On average, how often have you eaten the following food products over the last 12 months?
(Please tick one box per product group)

**In the last 12 months**	**Several times a day**	**Once a day**	**Several times a week**	**Once a week**	**Less often than once a week**	**never**
Sausages and cold meat	☐	☐	☐	☐	☐	☐
Poultry (*not including sausages and cold meat)*	☐	☐	☐	☐	☐	☐
Other meat (pork, beef, lamb) *(not including sausages and cold meat)*	☐	☐	☐	☐	☐	☐
Whole grain bread, other whole grain foods *(e.g. muesli)*	☐	☐	☐	☐	☐	☐
Fruit	☐	☐	☐	☐	☐	☐

10. On average, how many hours per week have you been physically active over the last 12 months?

In as far possible, please differentiate between light work, physically strenuous work and sporting activities

If you were not physically active (i.e. did not participate in sporting activities), please enter a zero.

Over the last 12 months

Physically strenuous work
(e.g. work in the agricultural and construction industries, or as a nurse for the sick or elderly…)

|_|_| hours per week *(on average)*

Physically strenuous sporting activities

(e.g. football, swimming, skiiing, challenging cycling, mountain climbing, jogging …)

|_|_| hours per week *(on average)*

Light work, conducted at walking speed

(e.g. housework, garden work, work as salesperson…)

|_|_| hours per week *(on average)*

Easy activities

(e.g. walking to work / going shopping, going for a walk, cycling…)

|_|_| hours per week *(on average)*

Thank-you for your participation
