# Supplementary material for: Positive family history of colorectal cancer in a general practice setting [FRIDA.Frankfurt]: study protocol of a of a cross-sectional study
Source: BMC Cancer. 2015 Aug 28;15:605. doi: 10.1186/s12885-015-1600-7 (PMC4552264; doi:10.1186/s12885-015-1600-7)
Supplement: Additional file 5: — Questionnaire 4. (PDF 225 kb) [file 12885_2015_1600_MOESM5_ESM.pdf]

Questionnaire 4

(The following boxes were filled out at the trial coordination center)

**Date**	
**Patient-ID**	

Dear participant,

Thank-you very much for taking the time to answer the following questions. The questionnaire will only take a few minutes to fill in. Please observe the following instructions:

- Please clearly tick the appropriate box. If you make a mistake, cross out the corresponding box and tick the correct one.
- **Please answer every question.** If you are in any doubt, you should tick the box which is most appropriate.
- Your answers will of course be treated in **strict confidence**. Only pseudonymized data will be used in the analysis.

If you have any questions, please do not hesitate to contact us. You will find contact details below. **Thank-you!**

1. Following the consultation with your family doctor, did you participate in bowel cancer screening by having an occult blood test?

☐ Yes

☐ No

2. Following the consultation with your family doctor, did you participate in bowel cancer screening by having a colonoscopy?

☐ Yes

☐ No

Please answer one of the following questions, depending on whether you participated in bowel cancer screening or not

If you underwent bowel cancer screening

3a. I regret having undergone bowel cancer screening.

☐ Yes

☐ No

If you did not undergo bowel cancer screening

3b. I regret not having undergone bowel cancer screening.

☐ Yes

☐ No

Questions about your thoughts and feelings

4. Over the last two weeks, how often have you been bothered by any of the following problems?

Please tick the appropriate answer with „✓"

Over the last two weeks	Not at all	Several days	More than half the days	Nearly every day
1. Feeling nervous, anxious or on edge	0	1	2	3
2. Not being able to stop or control worrying	0	1	2	3

5. Over the last two weeks, how often have you been bothered by any of the following problems?

Please tick the appropriate answer with „✓"

Over the last two weeks	Not at all	Several days	More than half the days	Nearly every day
1. Little interest or pleasure in doing things	0	1	2	3
2. Feeling down, depressed, or hopeless	0	1	2	3
3. Trouble falling or staying asleep, or sleeping too much	0	1	2	3
4. Feeling tired or having little energy	0	1	2	3
5. Poor appetite or overeating	0	1	2	3
6. Feeling bad about yourself – or that you are a failure or have let yourself or your family down	0	1	2	3
7. Trouble concentrating on things, such as reading the newspaper or watching television	0	1	2	3
8. Moving or speaking so slowly that other people could have noticed? Or the opposite – being so fidgety or restless that you have been moving around a lot more than usual?	0	1	2	3
9. Thoughts that you would be better off dead, or of hurting yourself in some way	0	1	2	3

Thank-you for your participation

Sources:

Frage 4: GAD-2: Kroenke K, Spitzer RL, Williams JBW. The PHQ-9. Validity of a brief depression severity measure. J Gen Intern Med 2001; 16: 606–613

Frage 5: PHQ-9:Kroenke K, Spitzer RL, Williams JBW, Monahan PO, Löwe B. Anxiety disorders in primary care: prevalence, impairment, comorbidity, and detection. Ann Intern Med 2007; 146: 317-325
